# Essential Oils of *Duguetia* Species A. St. Hill (Annonaceae): Chemical Diversity and Pharmacological Potential

**DOI:** 10.3390/biom12050615

**Published:** 2022-04-21

**Authors:** Albert C. dos Santos, Mateus L. Nogueira, Felipe P. de Oliveira, Emmanoel V. Costa, Daniel P. Bezerra

**Affiliations:** 1 Department of Chemistry, Federal University of Amazonas (UFAM), Manaus 69080-900, AM, Brazil; albertcdsantos@gmail.com; 2 Gonçalo Moniz Institute, Oswaldo Cruz Foundation (IGM-FIOCRUZ/BA), Salvador 40296-710, BA, Brazil; mateus.ln92@gmail.com (M.L.N.); felipepessoabiotec@gmail.com (F.P.d.O.)

**Keywords:** *Duguetia*, essential oil, chemical composition, pharmacological activity

## Abstract

*Duguetia* A. St. Hill (Annonaceae) is recognized as one of the major genera with approximately 100 species, 67 of which are found in Brazil (29 of those are endemic). They are arboreal species with edible fruits known as “pindaíba”, “pindaíva” “pinha”, and “envira” in Brazil. Many *Duguetia* species, in particular, have been used in traditional medicine to treat renal colic, stomachache, rheumatism, cough, toothache, muscle pain, fever, gastrointestinal pain, and breathing difficulties. In this study, we reviewed the chemical constituents and pharmacological properties of essential oils (EOs) from *Duguetia* species. A total of 12 species were found, along with their EO chemical constituents and bioactivities. Bicyclogermacrene, humulene epoxide II, spathulenol, germacrene D, caryophyllene oxide, viridiflorene, α-pinene, β-caryophyllene, and β-pinene were the main chemical constituents reported. The pharmacological effects of *Duguetia* species EOs included anti-inflammatory, antinociceptive, antibacterial, antifungal, antioxidant, anti-trypanosoma, cytotoxic and antitumor properties. This information adds to our understanding of the potential of the EOs of *Duguetia* species.

## 1. Introduction

The Annonaceae family is one of the largest in the Magnolalies order, with approximately 112 genera and 2440 species [1]. There are 32 genera and 392 species reported in Brazil, with three genera and 162 species being endemic [2]. The Amazon biome contains the greatest diversity of Annonaceae, with 27 genera and 280 species [2,3]. The main feature of this family is the availability of edible fruits, particularly those of the *Annona* and *Duguetia* species: *Annona squamosa* L. (sugar apple); *Annona mucosa* Jacq. (wild sweetsop); *Annona muricata* L. (soursop); *Duguetia lanceolata* A. St.-Hill (pindaba); and *Duguetia furfuracea* (cherry sugarapple) [1,2,3,4,5].

*Duguetia* A. St. Hill., along with *Guatteria* and *Annona*, is one of the largest genera in the Annonaceae family. This genus is found throughout the neotropics, including South America (from Nicaragua to Brazil) and the African Atlantic coast. Its trees or shrubs have flowers in the upper part of the trunk or long branches close to the ground from the trunk’s base. Leaves are adistichous with a midrib impressed on the upper side; flowers range from white to yellow. Fruits are pseudosyncarpous (carpels strongly coherent, but not or only partly connate), composed of 5 to over 500 carpels, with seeds (dark brown, obovoid, smooth, not arillate) embedded in a fleshy pulp [4,5]. 

Currently, the *Duguetia* genus contains approximately 100 species, 67 of which are found in Brazil and 29 of which are considered endemic [6,7,8,9]. Several *Duguetia* species have edible fruits with high economic value in Brazil, which are popularly known as “pindaíba”, “pindaíva”, “pinha”, and “envira” [4,5]. In terms of phytogeography distribution, 45 species are mostly found in the Amazon biome, while 17 are found in the Atlantic Forest [8].

Many species in the *Duguetia* genus are widely used to treat diseases such as renal colic, stomachache, rheumatism, cough, toothache, muscle pain, fever, gastrointestinal pain, and breathing difficulties [6,10,11,12,13,14,15,16,17]. The species used in traditional medicine are summarized in Table 1. On the other hand, while many ethnopharmacological uses for *Duguetia* species have been reported, only a few studies involving the chemical and therapeutic properties of this genus’ plants have been found.

According to Perez and Cassels [6], the chemical composition of this genus includes secondary metabolites, particularly alkaloids, found in various parts of *Duguetia* species. Furthermore, numerous studies have revealed the presence of steroids, flavonoids, aromatic compounds and terpenes. However, knowledge of the chemical composition and biological activities of this genus’ essential oils (EOs) is limited.

To guide future research, we discuss the chemical constituents and pharmacological properties of EOs of *Duguetia* species in this review. Google Scholar, PubMed, Science Direct, SciFinder, Scopus and Web of Science were used for searches. The following keywords were used: “Annonaceae”; *Duguetia*”; “essential oil”; “volatile constituents”; “volatile compounds”; and “biological activity”. A total of 82 articles were identified and reviewed, with those that included chemical constituents and biological activities of EOs being chosen and discussed.

## 2. Chemical Constituents of *Duguetia* Species EOs

EOs are natural, complex and volatile mixtures that can contain from 20 to 60 components in varying concentrations, with two or three main components having relatively high concentrations (20–70%) when compared to others that are present in small amounts, as well as having a strong odor and being composed of secondary metabolites [18,19]. The extraction of EOs is achieved through hydrodistillation or dragging of plant material with water vapor, and they are derived from various parts of aromatic plants (flowers, leaves, stem, branches, bark, wood, seeds and fruits), which are generally found in temperate- to hot-climate countries. They are well known for being liquid, clear, volatile, rarely colored, and soluble in lipids and organic solvents with densities less than water [20]. Furthermore, the chemical composition and biological activities of EOs can vary depending on the season, geographic area, climate, soil conditions, water stress, nutrition, isolation, or other abiotic factors [21,22].

**Figure 1:**
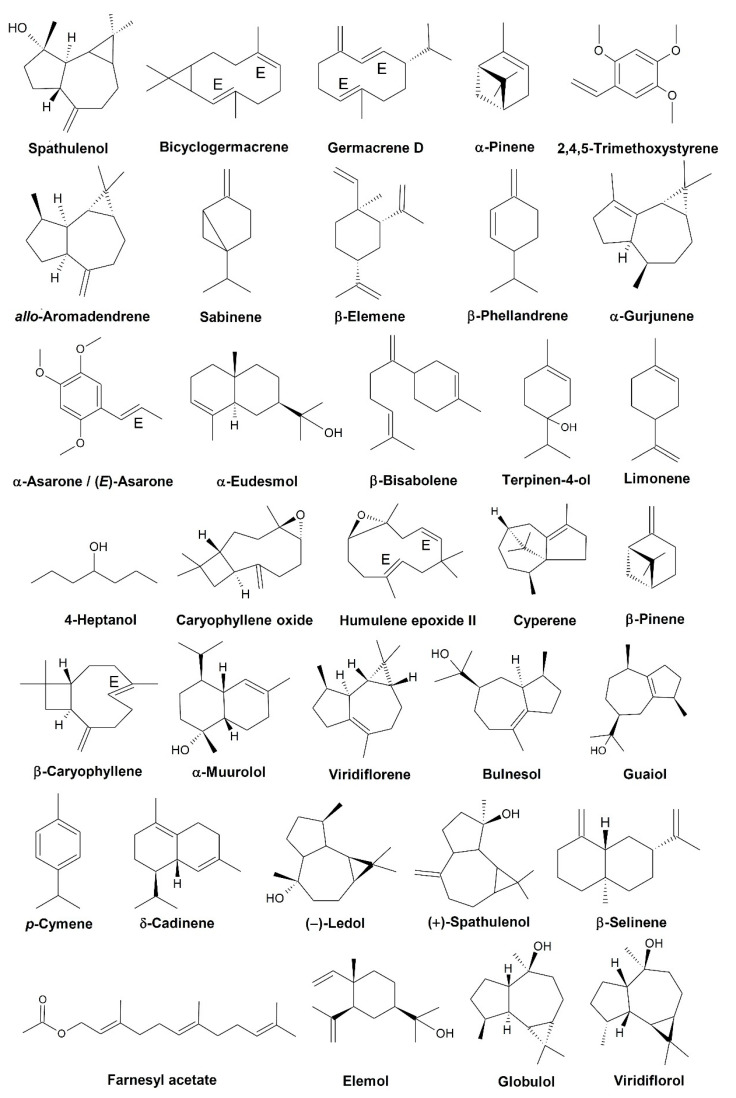
Chemical structures of the main constituents of the essential oils of *Duguetia* species.

Table 2 lists the main chemical constituents of EOs of *Duguetia* species. In total, the chemical composition of 56 constituents found in EOs from 12 species of this genus has been reported. These were *D. asterotricha* (Diels) R. E. Fries, *D. eximia* Diels, *D. flagellaris* Huber, *D. furfuracea* (A. St.-Hil.) Benth. & Hook. f., *D. gardneriana* Mart., *D. glabriuscula* (R. E. Fries) R. E. Fries, *D. lanceolate* A.St.-Hil., *D. moricandiana* Mart., *D. pycnastera* Sandwith, *D. quitarensis* Benth., *D. riparia* Huber, and *D. trunciflora* Maas. Figure 1 shows the structures of the major compounds identified in the EOs of these species.

Terpenes were the most identified among the main constituents that have been reported. Monoterpenes (11 components), hydrocarbon sesquiterpenes (21 components), oxygenated sesquiterpenes (16 components), and other groups (eight components), including alcohol and phenylpropanoid, were also reported. The terpenes with the most records were spathulenol and germacrene D (six species), α-pinene and β-caryophyllene (five species), caryophyllene oxide (four species) and, finally, bicyclogermacrene, humulene epoxide II, viridiflorene, β-phellandrene and β-pinene (three species). When comparing the constituents of EOs of the *Duguetia* genus and the Annonaceae family, some are highlighted among the genera of this family (*Annona, Anaxagorea, Fusae, Xylopia, Guatteria, Hexalobus,* and *Pachypodanthium*) as chemophenetic markers [37], namely bicyclogermacrene, spathulenol, germacrene D, caryophyllene oxide, α-pinene, and β-caryophyllene [38,39,40].

Spathulenol is the main component identified in EOs from the stem and leaves of *D. flagellaris* [24], *D. pycnastera* [24] and *D. riparia* [24], as well as in EOs from the leaves of *D. furfuracea* [27] and the branches of *D. flagellaris* [25]. This same constituent is found in EOs extracted from the stem and leaves of *D. eximia* [24], *D. furfuracea* [26,29] and *D. trunciflora* [24].

Valter et al. [29] identified bicyclogermacrene as the major constituent in the EOs from the leaves of *D. furfuracea*, whereas it was only a minor component in EOs from the stem of *D. furfuracea* [17,26,28], *D. pycnastera* and *D. trunciflora* [24], and in EOs from the leaves of *D. furfuracea* [27,29], *D. pycnastera* and *D. trunciflora* [24].

Germacrene D has been identified as a major component in EOs extracted from the leaves of *D. gardneriana* and *D. moricandiana* [30], as well as the bark and stem of *D. flagellaris* [24,25]. However, it was discovered as a minor constituent in the EOs from the leaves of *D. furfuracea* [29], *D. gardneriana* [31] and *D. pycnastera* [24], as well as the EOs from the stem of *D. pycnastera* [24] and aerial parts of *D. quitarensis* [36].

In *D. trunciflora*, α-pinene was found to be the most abundant constituent in stem and leaf EO samples [24]. On the other hand, it was not considered the major compound in EOs from the leaves of *D. gardneriana* [30], *D. moricandiana* [30] and *D. riparia* [24]. Although α-pinene was found in the EOs extracted from the flowers of *D. asterotricha* [23] and the stem of *D. riparia* [24], it was in a lower proportion. The 2,4,5-trimethoxystyrene identified in *D. furfuracea* [26] was described as the main component in the EO from the stem; however, it was present in lower concentrations in the EOs from the leaves of *D. lanceolata* [34] and the stem of *D. furfuracea* [17,27,28].

*Allo*-aromadendrene is the main constituent in the EO from the leaves of *D. gabriuscula*, as reported by Siqueira et al. [32]; however, it was found as a minor constituent in the EO samples from the stem and leaves of *D. pycnastera* [24]. Sabinene is the component with the highest percentage in EO extracted from *D. furfuracea* leaves [29]. β-Elemene is the most prevalent constituent in EO samples from bark and branches of *D. lanceolata* [33,35], but it is considered a minor constituent in the EO from the bark of *D. flagellaris* [24].

β-Phellandrene was found in high concentrations in the EOs extracted from the leaves of *D. furfuracea* [29] and the bark of *D. trunciflora* [24]. Similarly, α-gurjunene was identified as a major constituent in EO extracted from the stem of *D. furfuracea* [27]. This component has been found in lower concentrations in other studies of EO samples from the same species’ stem [17,28].

α-Asarone was found as a major constituent in EO from the stem of *D. furfuracea* [17], whereas no similar results were found in other studies of stem EO samples from the same species [26,27]. According to Maia et al. [24], α-eudesmol was the main component in EO samples from the leaves and stem of *D. eximia*, whereas β-bisabolene was the main compound identified in the EOs from the leaves of *D. gardneriana* [31] and *D. lanceolata* [34]. Terpinen-4-ol, limonene, 4-heptanol and (*E*)-asarone were found in the highest concentrations in the EOs extracted from *D. furfuracea* leaves [29], *D. asterotricha* flowers [23], *D. quitarensis* aerial parts [36] and *D. furfuracea* stem [28], respectively.

Caryophyllene oxide was found in the EOs of *D. flagellaris* bark [24], *D. furfuracea* leaves [29], *D. lanceolata* bark and branches [33,35], and *D. riparia* leaves and stem [24]. Humulene epoxide II was found in the leaves and stem EOs of *D. flagellaris* and *D. trunciflora* [24]. This component can also be found in EOs derived from the bark of *D. flagellaris* [24] and *D. lanceolata* [33]. 

Cyperene was identified as a chemical component in EOs extracted from the stem of *D. flagellaris* [25] and *D. furfuracea* [17,27,28], and *D. flagellaris* bark [24]. Whereas Almeida et al. [30] discovered β-pinene in the EOs extracted from the leaves of *D. gardneriana* and *D. moricandiana*. This last constituent can also be found in EOs derived of *D. trunciflora* leaves and stem [24].

β-caryophyllene and its synonyms *trans*-caryophyllene and (*E*)-caryophyllene have been reported in EOs of *D. furfuracea* leaves and stem [27,28,29], *D. gardneriana* and *D. moricandiana* leaves [30], *D. lanceolata* branches [35], and *D. quitarensis* aerial parts [36]. The presence of α-muurolol in the EOs from the bark, branches and stem of *D. flagellaris* has been reported [24,25]. Viridiflorene was found in EOs from the leaves of *D. gardneriana* and *D. moricandiana* [30], whereas Siqueira et al. [32] found it in EO from the leaves of *D. gabriuscula*.

Bulnesol and guaiol have been identified in EOs from the bark, leaves and stem of *D. trunciflora* [24]. *p*-Cymene was found as a minor constituent in EOs of *D. asterotricha* flowers [23] and *D. furfuracea* leaves [29], while δ-cadinene was found in EOs of *D. flagellaris* bark [24] and *D. lanceolata* branches [35]. Siqueira et al. [32] found (–)-ledol, (+)-spathulenol and farnesyl acetate in *D. gabriuscula* leaf EO.

β-Selinene was detected in EOs extracted from the bark and branches of *D. lanceolata* [33,35]. Elemol was identified in EOs extracted from the leaves and stem of *D. pycnastera* [24]. Globulol was discovered in EOs extracted from the leaves and stem of *D. trunciflora* [24]. Viridiflorol was found in EOs extracted from the leaves of *D. furfuracea* [27] and *D. gabriuscula* [29].

Some specific constituents are found in EOs from only a few species of the *Duguetia* genus, including: α-muurolene in the *D. flagellaris* bark EO [24]; *epi*-globulol in *D. furfuracea* stem EO [26]; and myrcene and α-phellandrene in *D. furfuracea* leaf EO [29].

A similar report has been made for *D. lanceolata*, in which the constituents *trans*-muurola-4(14),5-diene and 3,4,5-trimethoxystyrene are present in the EO from the leaves [34], whereas in the branch EO, cadina-1,4-dien-3-ol, β-eudesmol and δ-elemene are found [35]. Bay et al. [36] identified α-copaene and α-thujene as components of EO from the aerial parts of *D. quitarensis*. Maia et al. [24] discovered (*Z*)-β-farnesene, 7-*epi*-sesquithujene and α-cadinol in *D. trunciflora* bark EO. *Allo*-aromadendra-14β-al was discovered in the EO of *D. gabriuscula* leaves [32].

*Trans*-*m*-mentha-4,8-diene was identified in EOs extracted from the stem of *D. furfuracea* [27], elemicin in the leaves of *D. gardneriana* [31], δ-cadinene in the leaves of *D. furfuracea* [29], and β-sinensal and khusinol in the bark of *D. lanceolata* [33].

## 3. Pharmacological Properties of *Duguetia* Species EO

Natural products’ medicinal capabilities have historically been recognized in the most diverse cultures, allowing for widespread use in traditional folk medicine [41]. These findings have piqued the interest of the scientific community, which has been looking for novel drugs in medicinal plants for decades. As a result, new molecules with biological activities are being discovered, investigated, and converted for clinical use at an increasing rate [42].

In this study, we discovered that the most commonly reported actions of *Duguetia* species EOs are anti-inflammatory, antinociceptive, antimicrobial, antioxidant and cytotoxic activities. The pharmacological properties of *Duguetia* species EOs are summarizes in Table 3.

### 3.1. Anti-Inflammatory Activity

In the carrageenan-induced edema assay in the paws of Wistar rats, the anti-inflammatory activity of *D. lanceolata* bark EO was demonstrated [43]. The edema volume was significantly reduced when tested at doses of 50, 100 and 200 mg/kg, corresponding to reductions of 20.83%, 36.46% and 48.96%, respectively. Following that, *D. lanceolata* branch EO was tested in carrageenan-induced paw edema and pleurisy in rats as well as in mouse models of acute ear inflammation caused by croton oil and arachidonic acid [35]. In this case, EO (200 mg/kg), which contains the sesquiterpenes β-elemene (8.3%), caryophyllene oxide (7.7%) and β-eudesmol (7.2%) as main components, reduced paw edema by 44.1% while also significantly reducing carrageenan-induced exudate volume (50%) and the number of leukocytes (32.2%) in the pleurisy of the animals. In a dose-dependent manner, EO also significantly reduced the ear edema induced by arachidonic acid and croton oil.

Similarly, EO from the stem of *D. furfuracea*, with major components (*E*)-asarone (21.9%) and bicyclogermacrene (16.7%), was evaluated in Swiss mice [28]. Lipopolysaccharide (LPS)-induced paw edema was inhibited by 10 mg/kg oral treatment with values greater than 86%. With the same dose, EO reduced TNF-α production and polymorphonuclear-cell recruitment, and increased the expression of inducible nitric-oxide synthase (iNOS) in the paw tissue. All of these effects were amplified by a phenylpropanoid-enriched fraction from *D. furfuracea* EO, with the main constituents being α-asarone (36.4%) and 2,4,5-trimethoxystyrene (27.8%) [17]. In this study, EO at doses of 3 and 10 mg/kg inhibited paw edema by 90.91% and 92.42%, respectively, 2 h after LPS injection. After 4 h, a significant reduction effect was also observed with percentages of 77.78% (3 mg/kg) and 81.48% (10 mg/kg).

### 3.2. Antinociceptive Activity

The antinociceptive properties of EO derived from *D. lanceolate* and *D. furfuracea* have been studied. Sousa et al. [44] demonstrated that *D. lanceolata* bark EO was effective in the acetic-acid-induced abdominal-contraction assay and in the reduction in time of paw licking induced by formalin, attributing the positive results to the probable action of the EO components on the central and peripheral nervous systems. In the acetic-acid-induced writhing test, an effective dose of 50% (ED_50_) of 21.79 mg/kg was found, with a significant antinociceptive effect ranging from the dose of 10 mg/kg to total inhibition of writhing at 200 mg/kg. Paw-licking time was also reduced in a dose-dependent manner, with an ED_50_ of 5.27 mg/kg in the first phase (neurogenic phase) and 1.43 mg/kg in the second phase (inflammatory phase) [43].

*D. lanceolata* branch EO was also evaluated. After administration of EO at 100 and 200 mg/kg, there was a significant inhibition of acetic-acid-induced abdominal contractions. EO reduced paw-licking time in the neurogenic and inflammatory phases at doses of 50, 100 and 200 mg/kg. After 60 and 90 min of treatment with *D. lanceolata* EO, the animals’ response time in the hot-plate assay significantly increased, with the antinociceptive capacity partially maintained even in the presence of an opioid antagonist. Finally, in the tail-immersion assay, treatment allowed for a significant increase in the duration of pain latency [35].

The antinociceptive effects of EO extracted from the stem of *D. furfuracea* were also studied at doses of 10 and 30 mg/kg [28]. Paw-licking time was significantly reduced in the neurogenic and inflammatory phases after treatment with both doses, with the highest dose inhibiting formalin-induced nociception by 45.62% and 34.17%, respectively. The opioid antagonist naloxone (5 mg/kg) quickly reversed this effect in both the primary and secondary phases. In the LPS-induced thermal-hyperalgesia model, EO administration increased reaction time, allowing for a longer-lasting antinociceptive response (greater than 6 h after LPS-induced nociception) than morphine administration (7.5 mg/kg). Its antinociceptive activity may be mediated by adenosinergic and opioidergic pathways, but the rota-rod test revealed no changes in mouse motor coordination after treatments with 3, 10 and 30 mg/kg of the EO [28].

Similarly, Saldanha et al. [17] demonstrated the antinociceptive capacity of the phenylpropanoid-enriched fraction of *D. furfuracea* EO. For doses of 30 mg/kg, formalin-induced nociception was inhibited in both phases, with inhibition indices of 44.06% in paw-licking time in the first phase and 39.84% in the second. The reaction time to the LPS-induced thermal-hyperalgesia model increased. These effects were mediated by adenosinergic- and opioidergic-receptor activation, but without causing losses in the coordinative motor capacity of the tested animals [17].

### 3.3. Antibacterial and Antifungal Activities

The antimicrobial properties of EO extracted from the leaves of *D. gardneriana* and *D. moricandiana* were tested using the standard gel-diffusion method against a panel of fungal and bacterial strains. The results showed that the EO had a low inhibitory capacity against the tested microorganisms. Total EO of *D. gardneriana* (100% concentration), with the main components germacrene D (28.1%), viridiflorene (24.0%) and β-pinene (12.6%), demonstrated action against *Staphylococcus aureus* and *Candida guilliermondii*. The total EO of *D. moricandiana*, which contained the main constituents germacrene D (44.3%), α-pinene (13.0%), and viridiflorene (9.3%), was only effective against *Staphylococcus aureus* and *Candida albicans*. The average diameter of the halos was 12 mm in all cases [30].

The chemical composition and biological activities of EO extracted from the bark of *D. lanceolata* were found to be dependent on the extraction time [33]. Its main constituents were β-elemene (12.7 and 14.9%), caryophyllene oxide (12.4 and 10.7%) and β-selinene (8.4 and 10.4%). The use of EO in concentrations of 5, 10 and 25 mg inhibited the growth zone of *Staphylococcus aureus*, *Streptococcus pyogenes*, *Escherichia coli* and *Candida albicans*. The measurement of minimum inhibitory concentrations (MIC), which ranged from 20 to 125 μg/mL among microorganisms, confirmed this finding.

Bay et al. [36] investigated the EO from aerial parts of *D. quitarensis*. When tested using the microdilution-plate method, the EO, which was primarily composed of 4-heptanol (33.8%), α-thujene (18.4%) and (*E*)-caryophyllene (14.4%), demonstrated high antibacterial activity against *Streptococcus mutans* and *Streptococcus pyogenes*, with an MIC of 37.5 μg/mL against both gram-positive microorganisms.

### 3.4. Trypanocidal Activity

The trypanocidal activity of EO derived from the aerial parts of *D. quitarensis* Benth against *T. cruzi* trypomastigote and amastigote forms has been reported. The inhibitory concentration of 50% (IC_50_) for the EO was calculated to be 0.26 ± 0.06 μg/mL, which is approximately four times lower than the IC_50_ for benznidazole. However, when compared to the IC_50_ of EO in L929 cells, the selectivity index was found to be low (2.1 fold) [36].

### 3.5. Antioxidant Activity

Oxidative damage has been linked to the development and maintenance of inflammation, as well as the progression of many diseases. As a result, the antioxidant capacity of plants is being extensively researched. Sousa et al. [35] discovered antioxidant activity in *D. lanceolata* branch EO. The EO concentration required to scavenge 50% (effective concentration of 50%: EC_50_) of DPPH (2,2-diphenyl-1-picrylhydrazyl) free radicals was 159.4 ± 1.2 μg/mL, which was lower than the Fe^3+^ reducing power (187.8 ± 0.6 μg/ mL). The β-carotene/linoleic-acid bleaching assay also demonstrated this EO’s ability to inhibit lipid peroxidation by 41.5 ± 2.4%.

### 3.6. Cytotoxic and Antitumor Activity

Cytotoxicity studies are an important part of the drug-development process because they quickly provide vital information that serves as the initial platform in the search for new therapies for diseases such as cancer [45,46]. Despite being common among Annonaceae species, few studies have looked into the cytotoxic capacity of *Duguetia* species EO or their main components [47].

Matos et al. [44] found that (+)-*allo*-aromadendran-10,14β-diol, an aromadendrene-type sesquiterpene derived from the *D. glabriuscula* leaves EO, had a significant cytotoxic effect on human larynx carcinoma (Hep2) cells, with an IC_50_ value of 11.6 ± 2.3 μg/mL. The cytotoxic and antitumor potential of the EO of *D. gardneriana* leaves, with main components β-bisabolene (80.99%), elemicin (8.04%), germacrene D (4.15%), and cyperene (2.82%), was demonstrated against B16-F10 (16.89 µg/mL), HepG2 (19.16 µg/mL), HL-60 (13.08 µg/mL) and K562 (19.33 µg/mL) cancer cell lines [31]. Tumor-growth-inhibition rates of 5.37 and 37.52% were observed in C57BL/6 mice inoculated with B16-F10 cells after daily treatment with EO at 40 and 80 mg/kg/day, respectively [31].

The EO obtained from dry and fresh leaves of *D. glabriuscula*, with predominance of aromadendrene-type sesquiterpenes in its constitution (61.3% and 59.0%, from fresh and dried leaves, respectively), demonstrated toxic activity against *Artemia salina* in brine shrimp lethality bioassay (lethal concentration of 50%: LC_50_ = 1.6 mg/mL) [32]. Silva et al. [26] found that EO derived from the stem of *D. furfuracea* was active against *A. salina*, with an LC_50_ value of 2.6 μg/mL. The EO derived from stem of *D. furfuracea* (α-gurjunene: 22.2%; 2,4,5-trimethoxystyrene: 19.7%; cyperene: 16.0%) was toxic activity against *A. salina*, with an LC_50_ value of 715.2 mg/cm^3^ [27].

The toxicity of EO obtained from the bark of *D. lanceolata*, with β-elemene (12.7 and 14.9%), caryophyllene oxide (12.4 and 10.7%) and β-selinene (8.4 and 10.4%) as major components, was demonstrated by a brine-shrimp (*A. salina*)-lethality bioassay, with LC_50_ values of 49.0 μg/mL and 60.7 μg/mL, corresponding to different time of extraction [33].

Finally, Sousa et al. [35] evaluated the acute toxicity of *D. lanceolata* branch EO in Swiss albino mice. After oral administration of up to 3 g/kg of EO, the 50% lethal dose was calculated and determined to be 2.9 g/kg for 48 h. This assessment enabled the safe estimation of pharmacological doses for future experiments.

### 3.7. Other Activities

Ribeiro et al. [34] demonstrated the antiaflatoxigenic and insecticidal properties of EO extracted of *D. lanceolata* leaves. The EO, which is primarily composed of β-bisabolene (56.2%) and 2,4,5-trimethoxystyrene (19.1%), inhibited radial growth of *Aspergillus flavus* CCT7638 (Ascomycota) and the production of aflatoxin B1 from this isolate in a concentration-dependent manner.

Finally, the insecticidal activity was demonstrated using a residual-contact bioassay, which revealed a promising lethal effect on the adult forms of *Sitophilus zeamais* and *Zabrotes subfasciatus* (LC_50_ = 457 mg/kg and 442 mg/kg, respectively). The EO also significantly reduced the number of individuals emerging from the treated samples (EC_50_ = 480 mg/kg in adult *S. zeamais*; EC_50_ = 119 mg/kg in F1 progeny *Z. subfasciatus*), as well as the number of *Z. subfasciatus* eggs per treated sample (EC_50_ = 118 mg/kg) [34].

## 4. Conclusions

**Figure 2:**
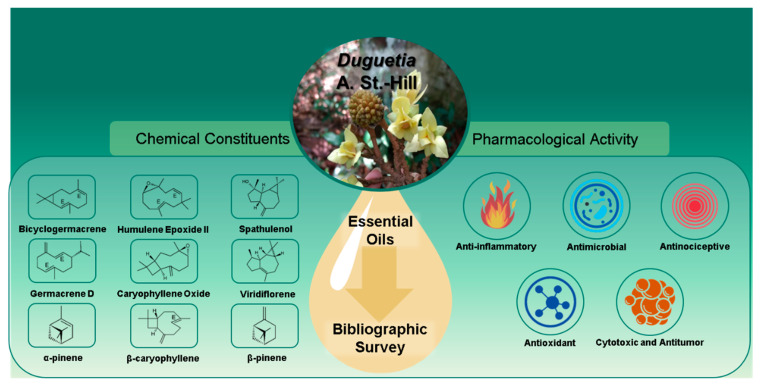
Main chemical composition and pharmacological properties of *Duguetia* species.

In recent years, studies on the genus *Duguetia* have focused on alkaloid constituents, resulting in few studies on EOs. As a result, this study discussed the chemical composition and pharmacological activities of the EOs of *Duguetia* species, demonstrating that this genus is a promising source of biologically active compounds. In this study, we discovered 56 chemical constituents found in 37 EOs of *Duguetia* species. The majority of the reported components are terpenes, specifically, monoterpenes, hydrocarbon and oxygenated sesquiterpenes. Bicyclogermacrene, humulene epoxide II, spathulenol, germacrene D, caryophyllene oxide, viridiflorene, α-pinene, β-caryophyllene and β-pinene are among these. The EOs of *Duguetia* species have been shown to have anti-inflammatory, antinociceptive, antibacterial, antifungal, antioxidant, anti-trypanosoma, cytotoxic, antitumor, antiaflatoxigenic and insecticidal properties. Figure 2 summarizes these findings. Further research into the chemical composition and pharmacological properties of the EOs of *Duguetia* species should be carried out in other species in order to assess their promising potential. In terms of future perspectives, mechanisms of action and toxicology studies should also be carried out in order to conduct clinical trials with these EOs.

## Figures and Tables

**Table 1:** Medicinal uses of *Duguetia* species.

Species	Popular Name in Brazil	Part Used	Popular Uses	References
*D. chrysocarpa*	Pindaíba-da-mata	Leaves; branch	Inflammatory diseases; gastrointestinal ulcers	[15,16]
*D. furfuracea*	Pinha do campo	Leaves	Renal colic	[17]
		Root	Stomachache	[11]
			Rheumatism	[12]
		Seeds	Against lice	[11]
*D. flagellaris*	Caniceiro preto	-	Rheumatism	[13]
*D. confinis*	Unknown	-	Cough; Toothache	[10]
*D. staudtii*	Unknown	Bark	Gastrointestinal pain; Breathing difficulties	[6]
*D. pycnastera*	Envira preta	Bark	Muscle pain	[14]
			Cough	
		Leaves	Fever	
			Cold sweat	

**Table 2:** Main chemical constituents of *Duguetia* species EOs.

Species (Popular Name in Brazil)	Part Used	Total Components	Major Constituents ^1^	References
*D. asterotricha* (envira, envira-surucucu-da-mata, envireira)	Flowers	9 (69.3%)	Limonene (14.1%), *p*-cymene (5.5%), and α-pinene (4.2%)	[23]
*D. eximia* (unknown)	Leaves and stem	76 (^4^)	α-Eudesmol (80.3%) and spathulenol (5.0%)	[24]
*D. flagellaris* (ameju-preto, caniceiro-preto, pindaíva, pindaíba)	Bark	76 (^4^)	Germacrene D (16.5%), cyperene (10.6%), α-muurolol (8.6%), humulene epoxide II (5.3%), spathulenol (5.0%), caryophyllene oxide (5.0%), δ-cadinene (4.3%), α-muurolene (4.2%), and β-elemene (4.0%)	[24]
	Stem	3 (^4^)	Germacrene D (16.5%), cyperene (10.6%) and α-muurolol (8.6%)	[25]
	Leaves and Stem	76 (^4^)	Spathulenol (58.7%), α-muurolol (6.2%) and humulene epoxide II (4.3%)	[24]
	Branches	2 (^4^)	Spathulenol (58.7%), and α-muurolol (6.2%)	[25]
*D. furfuracea* (araticum, ata brava, pinha do campo, pindaúva do campo, marolinho-do-cerrado, pinha-de-guará)	Stem	19 (^4^)	2,4,5-Trimethoxystyrene (29.2%), α-asarone (23.8%), bicyclogermacrene (8.6%), *epi*-globulol (6.4%), and spathulenol (4.7%)	[26]
		39 (^4^)	α-Gurjunene (22.2%), 2,4,5-trimethoxystyrene (19.7%), cyperene (16.0%), α-asarone (10.1%), and *trans*-*m*-mentha-4,8-diene (6.5%)	[27]
		24 (^4^)	(*E*)-Asarone (21.9%), bicyclogermacrene (16.7%), 2,4,5-trimethoxystyrene (16.1%), α-gurjunene (15.0%), cyperene (7.8%), and (*E*)-caryophyllene (4.6%)	[28]
		8 (^4^)	α-Asarone (36.4%), 2,4,5-trimethoxystyrene (27.8%), bicyclogermacrene (11.1%), α-gurjunene (10.5%), and cyperene (5.8%)	[17]
	Leaves ^2^	17 (99.4%)	β-Phellandrene (42.2%), bicyclogermacrene (20.7%), myrcene (6.8%), spathulenol (5.5%), α-phellandrene (4.6%), and sabinene (4.3%)	[29]
		17 (99.7%)	Sabinene (25.1%), terpinen-4-ol (16.2%), *p*-cymene (8.3%), caryophyllene oxide (7.7%), and spathulenol (5.1%)	
		18 (99.2%)	Bicyclogermacrene (29.1%), spathulenol (18.3%), germacrene D (9.6%), *trans*-caryophyllene (9.3%), δ-cadinene (5.5%), and caryophyllene oxide (5.3%)	
		18 (100%)	Bicyclogermacrene (24%), germacrene D (15%), trans-caryophyllene (12.9%), spathulenol (12.4%), and caryophyllene oxide (6.8%)	
		19 (100%)	Bicyclogermacrene (21.4%), germacrene D (13.6%), spathulenol (12.2%), and caryophyllene oxide (5.2%)	
		20 (99.7%)	Terpinen-4-ol (21.6%), spathulenol (20.9%), sabinene (17.3%), and p-cymene (5.6%)	
		31 (^4^)	Spathulenol (17.8%), bicyclogermacrene (16.2%), germacrene D (13.0%), β-caryophyllene (11.5%), and viridiflorol (4.0%)	[27]
*D. gardneriana* (jaquinha)	Leaves	33 (91.4%)	Germacrene D (28.1%), viridiflorene (24.0%), β-pinene (12.6%), α-pinene (9.1%), and β-caryophyllene (5.6%)	[30]
		4 (96%)	β-Bisabolene (81.0%), elemicin (8.0%), and germacrene D (4.2%)	[31]
*D. glabriuscula* (unknown)	Leaves	18 (^4^)	*allo*-Aromadendrene (22.6%), viridiflorene (13.3%), (–)-ledol (10.6%), α-santaleno (7.5%), (+)-spathulenol (5.8%), *allo*-aromadendrene-14β-al (5.0%), and farnesyl acetate (4.2%)	[32]
	Leaves	24 (^4^)	*allo*-Aromadendrene (16.2%), (–)-ledol (13.4%), (+)-spathulenol (12.1%), farnesyl acetate (5.9%), and viridiflorol (4.8%)	
*D. lanceolata* (araticum-bravo, pinha-brava, embireira, embira, pindaíba, pindaúba, pindaíva)	Bark ^3^	72 (99.6%)	β-Elemene (12.7%), caryophyllene oxide (12.4%), β-selinene (8.4%), humulene epoxide II (7.4%), and β-eudesmol (6.8%)	[33]
		51 (99.3%)	β-elemene (14.9%), caryophyllene oxide (10.7%) β-selinene (10.4%), β-eudesmol (7.9%), humulene epoxide II (6.8%), β-sinensal (5.4%), and khusinol (5.0%)	
	Leaves	5 (^4^)	β-Bisabolene (56.2%), 2,4,5-trimethoxystyrene (19.1%), *trans*-muurola-4(14),5-diene (12.2%), and 3,4,5-trimethoxystyrene (8.6%)	[34]
	Branches	37 (92.9%)	β-Elemene (8.3%), caryophyllene oxide (7.7%), β-eudesmol (7.2%), β-selinene (7.1%), β-caryophyllene (6.2%), δ-cadinene (5.5%), cadina-1,4-dien-3-ol (5.2%), cadalene (4.8%), and δ-elemene (4.1%)	[35]
*D. moricandiana* (unknown)	Leaves	33 (95.5%)	Germacrene D (44.3%), α-pinene (13.0%), viridiflorene (9.3%), β-pinene (9.2%), and β-caryophyllene (6.8%)	[30]
*D. pycnastera* (ata, envira, envira-preta, envira-surucucu)	Leaves and stem	76 (^4^)	Spathulenol (52.2%), *allo*-aromadendrene (9.1%), germacrene D (7.1%), elemol (5.1%), and bicyclogermacrene (4.8%)	[24]
*D. quitarensis* (ameju)	Aerial parts	20 (97.3%)	4-Heptanol (33.8%), α-thujene (18.4%), (*E*)-caryophyllene (14.4%), germacrene D (6.3%), and α-copaene (5.3%)	[36]
*D. riparia* (araticu da mata, envira-preta, makahymyra)	Leaves and stem	76 (^4^)	Spathulenol (46.5%), caryophyllene oxide (28.9%), and α-pinene (6.1%)	[24]
*D. trunciflora* (envireira, envira, invira)	Leaves and stem	76 (^4^)	α-Pinene (21.1%), bicyclogermacrene (17.6%), bulnesol (10.6%), spathulenol (10.5%), guaiol (8.1%), globulol (5.7%), humulene epoxide II (5.0%), and β-pinene (4.2%)	[24]
	Bark	76 (^4^)	β-Phellandrene (45.7%), guaiol (8.3%), α-cadinol (7.4%), (*Z*)-β-farnesene (4.8%), 7-*epi*-sesquithujene (4.5%), and bulnesol (4.2%)	

^1^ Only compounds with percentage concentrations greater than or equal to 4% are displayed. ^2^ The chemical compositions of the leaves vary due to the intensity of the odor and the location from which they were collected. ^3^ The extraction times for EO obtention (2 h and 4 h) resulted in different chemical compositions. ^4^ Percentage not described by the authors in relation to the total composition of the essential oil.

**Table 3:** Pharmacological properties of *Duguetia* species EOs.

Pharmacological Effects	Part Used	Actions	References
**Anti-inflammatory**			
*D. furfuracea*	Stem	After 6 h, EO inhibited paw edema induced by LPS by 92.4%.	[28]
	Stem	After 2 h of LPS injection, doses of 3 and 10 mg/kg of EO inhibited paw edema by 90.9% and 92.42%, respectively. After 4 h, there was a significant reduction effect, with percentages of 77.8% (3 mg/kg) and 81.5% (10 mg/kg).	[17]
*D. lanceolata*	Bark	EO at doses of 50, 100 and 200 mg/kg significantly reduced paw edema caused by carrageenan in 20.8%, 36.5% and 49.0%, respectively.	[43]
	Branches	After 4 h, EO reduced the formation of paw edema caused by carrageenan by 18.3% (50 mg/kg), 32.3% (100 mg/kg) and 44.1% (200 mg/kg).	[35]
**Antinociceptive**			
*D. furfuracea*	Stem	EO inhibited formalin-induced activity, and caffeine (10 mg/kg) and naloxone (5 mg/kg) administration reversed the EO’s antinociceptive activity.	[28]
	Stem	Inhibition of 43.4% and 44.1% of formalin-induced activity was observed during the 1st phase at doses of 10 and 30 mg/kg, respectively. In the 2nd phase, there was also reduction in licking time at doses of 10 mg/kg (30.9%) and 30 mg/kg (39.8%).	[17]
*D. lanceolata*	Bark	Number of abdominal contractions (ED_50_ = 21.8 mg/kg) and paw-licking time 1st phase (ED_50_ = 5.3 mg/kg) and 2nd phase (ED_50_ = 1.4 mg/kg) were reduced in the formalin test.	[43]
	Branches	In the formalin test, EO caused significant and time-dependent inhibition of paw licking at doses of 50, 100 and 200 mg/kg at 1st and 2nd phases.	[35]
**Antibacterial and Antifungal**			
*D. gardneriana*	Leaves	EO showed weak activity against *Staphylococcus aureus* and *Candida guilliermondii.*	[30]
*D. lanceolata*	Bark	EO inhibited the growth of *Staphylococcus pyogenes*, *Escherichia coli* and *Candida albicans* with MIC values ranging from 20 to 125 µg/mL.	[33]
*D. moricandiana*	Leaves	EO was active against *Staphylococcus aureus* and *Candida albicans*	[30]
*D. quitarensis*	Aerial parts	EO was active for Gram-positive microorganisms *Streptococcus mutans* and *Streptococcus pyogenes* with MIC of 37.5 µg/mL.	[37]
**Trypanocidal**			
*D. quitarensis*	Aerial parts	EO showed trypanocidal activity against the amastigote and trypomastigote forms of *Trypanosoma cruzi* with IC_50_ values of 0.26 and 0.54 µg/mL, respectively.	[36]
**Antioxidant**			
*D. lanceolata*	Branches	EO presented antioxidant effect, demonstrated through the DPPH radical, potency reduction and β-carotene assays. It inhibited lipid peroxidation by 41.5% (EC_50_ equal to 159.4 µg/mL).	[35]
**Cytotoxic and Antitumor**			
*D. furfuracea*	Stem	EO was active against *A. salina* with LC_50_ values of 2.6 µg/mL.	[26]
	Stem	EO was active against *A. salina* with LC_50_ of 715.2 mg/cm^3^_._	[27]
*D. lanceolata*	Bark	EO was cytotoxic against *A. salina* with LC_50_ of 49.0 and 60.7 µg/mL, corresponding to different times of extraction.	[36]
*D. gabriuscula*	Leaves	EO exhibited cytotoxicity towards tumor cell lines and showed IC_50_ value of 11.6 µg/mL for human larynx carcinoma (Hep2) cell line.	[44]
	Leaves	EO was toxic to *A. salina* with LC_50_ of 1.6 mg/mL.	[32]
*D. gardneriana*	Leaves	EO exhibited cytotoxic effect with IC_50_ values of 16.9, 19.2, 13.1 and 19.3 µg/mL against B16-F10, HepG2, HL-60 and K562 cell lines, respectively. In the in vivo experiment, tumor growth was reduced by 5.4 and 37.5% at doses of 40 and 80 mg/kg, respectively.	[31]

## Data Availability

Not applicable.

## Abbreviations

DPPH	2,2-diphenyl-1-picrylhydrazyl
EC_50_	effective concentration of 50%
ED_50_	effective dose of 50%
EOs	essential oils
IC_50_	inhibitory concentration of 50%
LC_50_	lethal concentration of 50%
LPS	lipopolysaccharides
MIC	minimum inhibitory concentration
